# A Systematic Review of the Efficacy and Safety of Fecal Microbiota Transplant for* Clostridium difficile* Infection in Immunocompromised Patients

**DOI:** 10.1155/2018/1394379

**Published:** 2018-09-02

**Authors:** Oluwaseun Shogbesan, Dilli Ram Poudel, Samjeris Victor, Asad Jehangir, Opeyemi Fadahunsi, Gbenga Shogbesan, Anthony Donato

**Affiliations:** ^1^Department of Medicine, Tower Health System, Sixth Avenue and Spruce Street, West Reading, PA 19611, USA; ^2^Hospitalist Services, Tower Health System, Sixth Avenue and Spruce Street, West Reading, PA 19611, USA; ^3^Department of Biochemistry & Molecular Biology, Pennsylvania State University, State College, PA 16801, USA; ^4^Division of Cardiology, Dalhousie University, Halifax, NS B3H 4RS, Canada; ^5^Department of Internal Medicine, Piedmont Athens Regional Medical Center, Athens, GA 30606, USA

## Abstract

**Background:**

Fecal microbiota transplantation (FMT) has been shown to be effective in recurrent* Clostridium difficile* (CD) infection, with resolution in 80% to 90% of patients. However, immunosuppressed patients were often excluded from FMT trials, so safety and efficacy in this population are unknown.

**Methods:**

We searched MEDLINE and EMBASE for English language articles published on FMT for treatment of CD infection in immunocompromised patients (including patients on immunosuppressant medications, patients with human immunodeficiency virus (HIV), inherited or primary immunodeficiency syndromes, cancer undergoing chemotherapy, or organ transplant, including-bone marrow transplant) of all ages. We excluded inflammatory bowel disease patients that were not on immunosuppressant medications. Resolution and adverse event rates (including secondary infection, rehospitalization, and death) were calculated.

**Results:**

Forty-four studies were included, none of which were randomized designs. A total of 303 immunocompromised patients were studied. Mean patient age was 57.3 years. Immunosuppressant medication use was the reason for the immunocompromised state in the majority (77.2%), and 19.2% had greater than one immunocompromising condition. Seventy-six percent were given FMT via colonoscopy. Of the 234 patients with reported follow-up outcomes, 207/234 (87%) reported resolution after first treatment, with 93% noting success after multiple treatments. There were 2 reported deaths, 2 colectomies, 5 treatment-related infections, and 10 subsequent hospitalizations.

**Conclusion:**

We found evidence that supports the use of FMT for treatment of CD infection in immunocompromised patients, with similar rates of serious adverse events to immunocompetent patients.

## 1. Introduction

*Clostridium difficile* (CD) infection is the leading cause of healthcare-associated diarrheal illness in the United States, affecting nearly 500,000 patients annually [1, 2]. Both incidence and severity of CD infection have increased over the past two decades, and CD infection is now responsible for 29,000 deaths/year within 30 days of diagnosis [1]. Immunocompromised patients, including those receiving immunosuppressant medications or patients with human immunodeficiency virus (HIV) and transplants, seem to be at increased risk of hospitalization and recurrence of CD infection as the immune system is an important defense for both protection and recovery from infection [3–6].

Antibiotics have long been the mainstay of treatment for CD infection. However, 25% of patients suffer recurrence of CD infection within 60 days of antibiotic therapy [7, 8]. FMT has emerged as an effective alternative for the relapsed and refractory CD infection patients with reported success rates of 80-90% in clinical trials [9, 10]. Due to safety concerns related to introducing bacterial therapy in immunocompromised patients, those with immunocompromised states have been excluded from most trials, and guidelines currently recommend caution in these patient populations due to the absence of safety and efficacy data [11, 12].

The aim of our study is to conduct a systematic review of the existing literature to collate the evidence for efficacy and safety of FMT in immunocompromised population.

## 2. Methods

We searched PubMed, EMBASE, and Google Scholar for English language articles published on FMT for treatment of CD infection from inception through May 2017.

These databases were searched using the search terms under 2 broad search themes of “*Clostridium difficile*” and “fecal microbiota transplantation” and were combined using a Boolean operator AND (see supplementary file 1). For the term “*Clostridium difficile*”, we used a combination of MeSH entry term words* Clostridium difficile* and* C. difficile*. For the MeSH term “fecal microbiota transplantation”, we used synonyms for fecal microbiota transplantation, intestinal microbiota transfer, donor feces infusion, and stool transplant. We made the decision not to include the term “immunocompromised” due to concerns that our search would not capture the patients broadly enough. We instead reviewed all individual articles for descriptions of treated patients who matched our definition of immunocompromised.

We defined a patient as immunocompromised if that patient was receiving immunosuppressive agents (including but not limited to mTOR inhibitors, calcineurin inhibitors, anti-TNF agents, other biologic agents, high dose steroids > 20 mg/day or ≥ 1 mg/kg for > 14 days), patients with human immunodeficiency virus (HIV) infection (regardless of CD4 count), acquired immune deficiency syndrome (AIDS), inherited or primary immunodeficiency syndromes, hematologic malignancy or solid tumor (active with treatment in past 3 months or in remission for less than 5 years), solid organ transplant, and/or bone marrow transplant. We excluded inflammatory bowel disease (IBD) patients that were not receiving immunosuppressant medications. We also excluded patient with chronic medical conditions such as chronic liver disease, chronic kidney disease, and autoimmune conditions not on immunosuppressant. We included patients of all age groups.

Our outcomes of interest were clinical resolution of diarrhea, bacteriologic resolution, treatment failure, adverse events, and mortality. Clinical or bacteriologic resolution was defined as absence of diarrhea or need for further CDI treatment after FMT within the study or follow-up period clinically or with C. difficile toxin testing, respectively. Treatment failure was defined as nonresponse or recurrence of diarrhea with or without positive C. difficile toxin. We defined post-FMT death as any death within 30 days of FMT.

We reviewed all study types with original data published in English language. The reference lists of included articles and chosen articles were manually hand-searched for additional articles. Our eligibility criteria for inclusion were as follows: (1) studies of any type on human subjects with a full published manuscript who met at least one of our definitions for immunocompromised, (2) received fecal transplant via any method for a laboratory-confirmed, symptomatic CD infection, and (3) any of the outcomes of interest was reported in the manuscript. We included patients who received FMT in inpatient, outpatient, or home setting. We excluded studies that evaluated FMT for non-CD illness. We excluded conference abstracts to avoid duplication of our study population with a subsequent full publication. We excluded studies that did not report on any of our outcomes or had mixed population of immunocompromised and immunocompetent patients that did not report outcomes of immunocompromised population separately.

Three reviewers (YF, SV, and OS) independently screened titles and abstracts and excluded irrelevant studies. Full manuscript review was conducted by three investigators (YF, SV, and OS) to determine inclusion eligibility. Disagreement on inclusion was adjudicated by a third investigator (AD). Data extraction was performed by 3 investigators (GS, AJ, and SV) and reviewed for accuracy by a third investigator (OS).

We extracted data on patient's characteristics including age, gender, number of CD infections prior to FMT, interventions prior to FMT, time from index CD infection diagnosis to FMT, method of diagnosis of index CD infection, and reasons for immune compromise. We collected study characteristics including study type, location, clinical setting, and duration of study including length of follow-up period. We also extracted FMT treatment data, including delivery method (upper GI infusion, capsule ingestion, colonoscopic infusion, or enema), number of treatments, whether fresh or frozen stool was administered, treatment dose infused, stool donor relationship (related or unrelated), pretransplant bowel preparation, and pretransplant use of antibiotics. Outcome data collected included resolution of clinical symptoms, treatment failure after single FMT, all-cause mortality within 30 days, number of relapses, and need for additional FMT prior to resolution. We also categorized adverse events including colectomy, CD/FMT-related deaths, new hospitalizations, life-threatening events, need for surgery, infection complications, IBD flares, and time from infection to adverse event. A CD/FMT-related adverse event was defined as any complication or new event occurring within 30 days of first FMT. Duplicate patient entries were identified and removed. Authors were contacted for clarification on data where necessary.

We assessed study quality using questions from the NIH quality assessment tool for case series studies. We conducted quality assessment only on studies with at least five patients in original study population (Supplementary file 2) [57].

We did pooled studies and calculated resolution and adverse event rates with 95% confidence interval using STATA version 13 (College Station, TX). We set statistical significance at p ≤ 0.05. Some studies reported adverse events but had missing data for efficacy. Given the importance of adverse event outcomes in immunocompromised patients, we conducted separate efficacy and safety analyses.

There were no randomized controlled trials and study heterogeneity between the nonrandomized trials precluded performing a meta-analysis on our included studies.

## 3. Results

**Figure 1:**
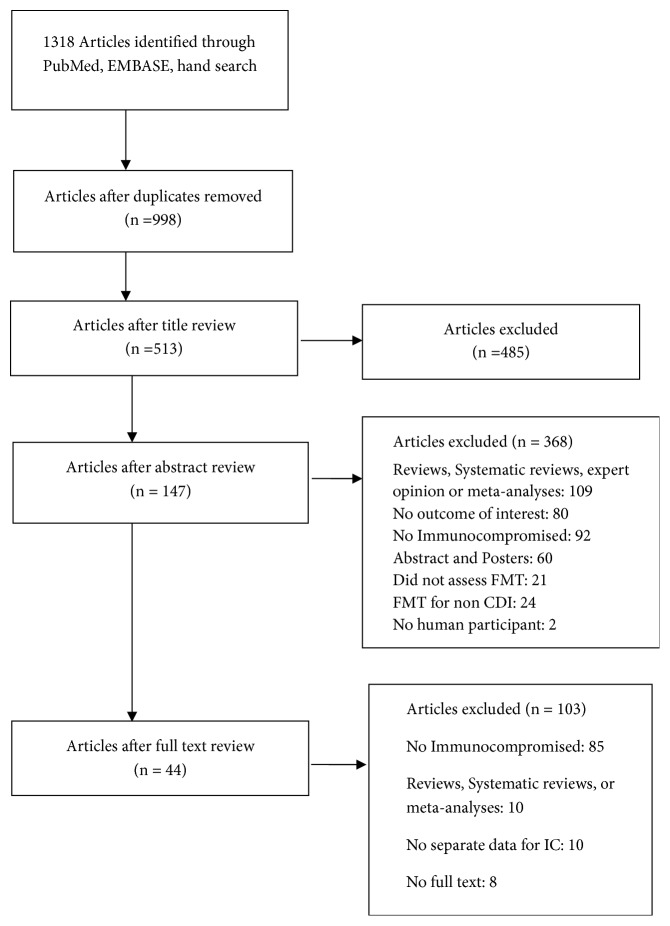
Flowchart for study selection.

We identified 44 studies which met inclusion criteria describing 303 patients (Figure 1) [13–56]. Forty-three were single cases or case series and one was a retrospective cohort study, and no randomized designs were identified (Table 1). Of those studies reporting gender, 62% were females and 38% were males. The mean age was 57.3 years (range: 2-88 years). The most common reason for the immunocompromised state was use of immunosuppressant medication (77.2%). Other reasons for being immunocompromised included solid organ transplant (18.2%), active malignancy including lymphoma or leukemia (16.2%), hematopoietic stem cell transplant (2.5%), and HIV/AIDS (2.1%). There was more than one immunocompromising condition in 19.9% of patients.

Patient averaged about 2.5 episodes of CD prior to first FMT. Most patients (73.7%) had received other treatments for CD infection, mainly antibiotics, before FMT, with many (48.6%) receiving 2 or more CD infection treatments prior to FMT. Treatments other than antibiotics prior to FMT included probiotics, intravenous immunoglobulin, and surgery. For patients that received antibiotics prior to FMT, antibiotics were stopped on average about 1.5 days (range: 0-3, SD: 0.55 days) prior to FMT procedure.

Colonoscopy was the route of delivery of FMT in 76% of patients, while 21% had stool transplanted via ingestion of capsules or other upper gastrointestinal route (nasal tubes or endoscopy). Retention enema was performed in 7.6 % of patients. Most patients (95%) received fresh stool, while 5% utilized commercially prepared products. Among those reporting source of stool, a related donor was employed in 75% of patients.

A total of 234 patients had data on outcome and were included in the efficacy analysis. Of these, 206 (87.7%) had clinical resolution of CD infection after first FMT treatment, while 93% had resolution after 2 or more FMT attempts. Comparing rate of resolution by delivery method, colonoscopy delivered FMT had an 84% success rate, while upper gastrointestinal delivery (via endoscopy, capsule, and nasogastric or nasojejunal tubes) resulted in 92% success rate (p = 0.202). In terms of number of immunocompromising conditions, patients with one condition had a success rate of 93%, while those with two or more immunocompromising conditions were resolved 78% of the time (Odds ratio (OR) 0.24, 95% CI: 0.11- 0.51, p<0.0001).

All 303 patients were included in the safety analysis. There were 2 reported deaths. Both deaths were in patients with solid organ transplants. One patient died 13 days after successful FMT, with death due to progressive pneumonia, while the second patient died 1 day after FMT following aspiration pneumonitis during sedation for colonoscopy. Other reported adverse events include 2 colectomies, 5 episodes of bacteremia or infection, 10 subsequent hospitalizations, 7 otherwise unspecified life-threatening complications, and 7 flares of inflammatory bowel disease. Twenty-eight patients had other complications including abdominal pain, irritable bowel syndrome, nausea, fever, and diverticulitis post-FMT procedure. Mean time to adverse event was 26.6 days (range: 0-56, SD: 34.3 days) from FMT (Table 2).

Twenty of the included 43 case reports/studies had at least 5 patients in the original study population. Only 10 studies showed adequate reporting in all of six essential domains of study quality (study objective, case definition, outcome measure definition, FMT procedure, adequacy of follow-up, and donor characteristics), with others missing 1 to 3 of these elements (Supplementary file 2).

## 4. Discussion

Our review identified an 88% success rate after a single FMT and 93% after multiple FMTs in our immunocompromised population, which parallels the 80-90% success rates reported in the general population [9, 10]. Patients with a single immunocompromising factor had a higher rate of treatment success when compared to patients with multiple immunocompromising factors (p<0.001). In comparison, a retrospective series by Kelly et al. looking at 80 immunocompromised patients with CD infection treated with FMT reported a 78% cure rate following a single FMT and 89% cure rate with multiple FMT [28]. Of these 80 patients, 38 met our inclusion criteria and were included in our analyses. A recent systematic review and meta-analysis by Ianiro et al. found a similar cure rate of 93% after multiple FMT with a 76% cure rate after a single FMT [58]. While Ianiro et al. excluded case reports and case series with less than 10 patients who received FMT for CDI with a minimum of 8 weeks follow-up, our study focused on only immunocompromised patients regardless of the study size given our already limited study population.

Safety concerns were the rationale for excluding immunocompromised patients from clinical trials and expression of caution in guidelines for FMT. We identified just 2 deaths among out 303 patients with 30 days of FMT. Both deaths were reported in a retrospective review by Kelly et al. but we could not directly ascertain whether those deaths were directly related to FMT, to the CD infection or the patient's underlying immunocompromised states. Other deaths in our included studies were either not related to FMT (post-colectomy complications) or occurred beyond 30 days after FMT [23, 37]. Of those reporting rehospitalization following FMT, 8.3% reported this. While fecal transplant has been associated with reactivation of existing immune-mediated disorders or new disorders such as immune thrombocytopenia, rheumatoid arthritis in immunocompetent patients following treatment, this side effect was not identified in our study [59]. It is possible that the underlying immunosuppressed states of our study population may have suppressed any adverse immunologic responses observed in immunocompetent patients.

Our study has the following strengths. It addresses a very specific population with CD infection that has a higher incidence of CD infection with higher risk of recurrence and would ideally benefit from FMT. In addition, we included only patients who met a standard, predetermined definition of immunosuppression. However, our study has some limitations. We reviewed case reports and series, as there were no RCTs that were identified for inclusion. Inclusion of case reports with possibility of publication bias towards positive results might account for the high success rate after a single FMT. Missing data on demographics, method of stool transplantation, volume and amount of stool, and relationships of donor and recipients were common in our review and were also noted in a similar review by Bafeta et al. [60]. One clinical trial had immunocompromised patients that met inclusion criteria but had a mixed population of patients that included immunocompetent patients and did not provide separate data on the included immunocompromised population and therefore could not be included in our study [61]. Our efforts at contacting authors to provide data on immunocompromised patients were unsuccessful. In the absence of clinical trials, overall studies were too heterogeneous precluding a meta-analysis.

## 5. Conclusion

In conclusion, FMT in immunocompromised appears to have comparable efficacy and safety data to those on patients with intact immunity. However, due to heterogeneity of immunosuppression subtype, no solid conclusion can be made about any single specific immunocompromised states or a combination regarding response to FMT. Further randomized trials including these patient populations would be appropriate.

## Figures and Tables

**Table 1:** Summary of included articles.

Author, Year	Article type	N patients included	Immunocompromised	FMT delivery	N transplant	Stool (g or volume (mL per transplant	Donor relationship	AE
Aas, 2003 [13]	Case series	1	Leukemia	NG Tube	1	30 g/25 ml	NR	No

Aratari, 2015 [14]	Case report	1	IBD on IS	Colonoscopy	1	150 g	UR	No

Bilal, 2015 [15]	Case report	1	Liver and kidney transplant on tacrolimus	Colonoscopy	1	180 ml	R	No

Blackburn, 2015 [16]	Case report	1	Leukemia	Colonoscopy	1	NR	NR	No

de Castro, 2015 [17]	Case Report	1	ALL s/p HSCT	Upper Endoscopy	1	NR	NR	No

Duplessis, 2012 [18]	Case report	1	CD on IS	UG Upper Endoscopy	1	75 g/200 ml	R	No

Ehlermann, 2014 [19]	Case report	1	Heart transplant	Upper Endoscopy	1	100 ml	R	No

Elopre, 2013 [20]	Case series	2	AIDS, DM + AIDS	Upper Endoscopy	1	30 g/25 ml	R	No

Fischer, 2016 [21]	Cohort	101	Multiple defined	Colonoscopy	1	NR	NR	NR

Friedman-Moraco, 2014 [22]	Case series	2	SOT	Upper Endoscopy and Colonoscopy	2	30 g/80-325 ml	R	Yes

Garborg, 2010 [23]	Case series	1	AML	Upper Endoscopy	1	50-100 g/200 ml	R	No

Gathe, 2016 [24]	Case report	1	HIV	Colonoscopy, Enema and Nasogastric tube	4	NR	R	No

Guiterrez-Delgado, 2016 [25]	Case series	1	Acute leukemia	Colonoscopy	2	NR	UR	No

Gweon, 2015 [26]	Case report	1	Thyroid cancer	Colonoscopy and Upper Endoscopy	1	75 g	UR	No

Hirsch, 2015 [27]	Case series	5	Lymphoma, AML, Renal cell CA, IS	Oral (ingested	1-4	2.3 g (6-22 capsules)	UR	Yes

Hourigan, 2015 [28]	Case series	3	IBD on IS	Colonoscopy	1	92 g	R	No

Kelly, 2014 [29]	Case series	46^*∗*^	IS, SOT, Severe/end stage chronic disease, Cancer, HIV	Colonoscopy^#^	1-2	NR	NR	Yes

Khoruts, 2016 [30]	Case series	38	SOT, IS	Colonoscopy	1	NR	NR	NR

Kronman, 2014 [31]	Case series	3	IBD on IS	NG Tube	1	30-60 ml	R	No

Laszlo, 2016 [32]	Case series	1	UC on IS	Colonoscopy	1	150 ml	R	Yes

Lee, 2014 [33]	Case report	1	Liver transplant	Upper Endoscopy	2	NR	R	Yes

Lee CH, 2014 [34]	Case series	3	Renal transplant	Enema	1-4	100 ml	UR	No

Loke, 2016 [35]	Case report	1	Specific Antibody Deficiency (SAD	Colonoscopy	1	50 g/500 ml	R	NR

Mandalia, 2016 [36]	Case series	37	HIV, AIDS, malignancy, IS	NR	1-3	NR	NR	Yes

Mattila, 2012 [37]	Case series	3	Lung transplant	Colonoscopy	1-2	100 ml	R and UR	No

Mittal, 2015 [38]	Case report	1	Diffuse large B cell non-Hodgkin's lymphoma +UC	Enema	2	NR	NR	Yes

Neemann, 2015 [39]	Case report	1	ALL s/p HSCT	Upper Endoscopy	1	30 ml	R	NR

Ott, 2017 [40]	Case series	3	Kidney transplant, HIV, Colon Cancer	Upper Endoscopy	1	NR	R and UR	No

Pathak, 2014 [41]	Case series	3	Adenocarcinoma left colon, Renal transplant, Cancer	Colonoscopy	1	6-8 teaspoons, 40- 500ml	R	No

Pierog, 2014 [42]	Case series	2	IBD on IS	Colonoscopy	1	60 ml	R	Yes

Porter, 2014 [43]	Case report	1	B cell CLL	Upper Endoscopy	6	50 g	UR	NR

Quera, 2013 [44]	Case report	1	CD on IS	Colonoscopy	1	NR	NR	Yes

Ramay, 2015 [45]	Case report	1	Heart transplant	Colonoscopy	2	250 ml	UR	No

Ray, 2014 [46]	Case series	2	Multiple	Colonoscopy	1	60 ml	R	No

Rubin, 2012 [47]	Case series	15	Malignant disease	Upper Endoscopy	1	30 g/25 ml	R	No

Russell, 2014 [48]	Case series	1	UC on IS	Colonoscopy	1	30-40 g/250 ml	R	Yes

Schunemann, 2013 [49]	Case report	1	AIDS	Colonoscopy and Upper Endoscopy	2	NR	R	No

Silverman, 2010 [50]	Case series	3	Lymphoma, liver transplant	Enema	1	50 ml	R	Yes

Stripling, 2015 [51]	Case report	1	Cardiac, kidney transplant on IS	Upper Endoscopy	1	NR	R	No

Trubiano, 2014 [52]	Case report	1	Diffuse large B cell lymphoma	Upper Endoscopy	2	260 ml	R	No

Webb, 2016 [53]	Case series	5	HSCT	NJ tube	1	25-100 g	NR	Yes

Weingarden, 2013 [54]	Case series	1	Metastatic Ovarian cancer	Colonoscopy	1	NR	UR	Yes

Yoon, 2010 [55]	Case series	2	Colon cancer, Breast cancer	Colonoscopy	1	NR	R	No

Zainah, 2012 [56]	Case report	1	UC on IS	Colonoscopy	1	300 ml	R	No

AE= adverse events; ALL= acute lymphocytic leukemia; AML= acute myeloid leukemia; CA= cancer; CD=Crohn's disease; CLL= chronic lymphocytic leukemia; E= enema; HIV= human immunodeficiency virus: IBD= inflammatory bowel disease: IS= immunosuppressant; HSCT= hematopoietic stem cell transplant; LTE= letter to editor; NG= nasogastric tube; NR= not reported or no separate patient level data on immunocompromised patients; R= related (genetically or household; SOT= solid organ transplant; UC= ulcerative colitis; UG= upper gastrointestinal endoscopy; and UR= unrelated

*∗*= more IC patients but only including those with separate data that we could match to IC status, outcome, and AE

#= article reports where most centers used colonoscopy; possibly some included patients had other route of delivery.

**Table 2:** Adverse events (AE) in immunocompromised patients with recurrent CD infection treated with FMT.

Author, Year	Patients with events (N)	Type of AE
Friedman-Moraco, 2014 [22]	1	Life threatening event: ischemic stroke
Hirsch, 2015 [27]	1	New Hospitalization
	1	Life threatening event
	1	Abdominal pain
Kelly, 2014 [29]	1	Colectomy
	1	Death
	1	Death
	5	New Hospitalization
	1	Life threatening event
	3	Infection: pneumonia, Influenza, Pertussis
	4	IBD flare
	11	Others:
		Hip pain
		Nausea
		Bloating
		Fever
		Diarrhea
		Abdominal pain
		Catheter infection
		Self-limited diarrhea
		Minor mucosal tear during colonoscopy
Laszlo, 2016 [32]	1	Others: Mild abdominal pain
Lee, 2014 [34]	1	New Hospitalization/Life threatening event
Mandalia, 2016 [36]	3	IBD flare
	1	Diverticulitis
Mittal, 2015 [38]	1	New Hospitalization
Pierog, 2014 [42]	1	Life-threatening event/New
		Hospitalizations/Surgery
Quera, 2013 [44]	1	Life threatening event
		Infection: Pan-sensitive *E. coli*
Russell, 2014 [48]	1	Colectomy/New Hospitalization/Life threatening events/Surgery
Silverman, 2010 [50]	3	IBS
Webb, 2016 [53]	5	Abdominal pain
Weingarden, 2013 [54]	1	Colectomy

AE= adverse event; IBS= irritable bowel syndrome.

## Supplementary Materials

Supplementary 1 Supplemental File 1: search strategy.

Supplementary 2 Supplemental File 2: study quality assessment using a modified NIH Quality Assessment Tool.

## References

[1] Lessa F. C., Winston L. G., McDonald L. C. (2015). Emerging infections program C. difficile surveillance team. Burden of Clostridium difficile infection in the United States. *The New England Journal of Medicine*.

[2] Drozd E. M., Inocencio T. J., Braithwaite S. (2015). Mortality, hospital costs, payments, and readmissions associated with clostridium difficile infection among medicare beneficiaries. *Infectious Diseases in Clinical Practice*.

[3] Kamboj M., Son C., Cantu S. (2012). Hospital-onset clostridium difficile infection rates in persons with cancer or Hematopoietic stem cell transplant: A C3IC network report. *Infection Control and Hospital Epidemiology*.

[4] Alonso C. D., Kamboj M. (2014). Clostridium difficile infection (CDI) in solid organ and hematopoietic stem cell transplant recipients. *Current Infectious Disease Reports*.

[5] Raza S., Baig M. A., Russell H., Gourdet Y., Berger B. J. (2010). *Clostridium difficile* infection following chemotherapy. *Recent Patents on Anti-Infective Drug Discovery*.

[6] Schneeweiss S., Korzenik J., Solomon D. H., Canning C., Lee J., Bressler B. (2009). Infliximab and other immunomodulating drugs in patients with inflammatory bowel disease and the risk of serious bacterial infections. *Alimentary Pharmacology & Therapeutics*.

[7] Kelly C. P., LaMont J. T. (2008). Clostridium difficile—more difficult than ever. *The New England Journal of Medicine*.

[8] McFarland L. V., Elmer G. W., Surawicz C. M. (2002). Breaking the cycle: Treatment strategies for 163 cases of recurrent Clostridium difficile disease. *American Journal of Gastroenterology*.

[9] van Nood E., Vrieze A., Nieuwdorp M. (2013). Duodenal infusion of donor feces for recurrent clostridium difficile. *The New England Journal of Medicine*.

[10] Cammarota G., Masucci L., Ianiro G. (2015). Randomised clinical trial: faecal microbiota transplantation by colonoscopy vs. vancomycin for the treatment of recurrent. *Alimentary Pharmacology & Therapeutics*.

[11] Bakken J. S., Borody T., Brandt L. J. (2011). Treating clostridium difficile infection with fecal microbiota transplantation. *Clinical Gastroenterology and Hepatology*.

[12] Sokol H., Galperine T., Kapel N. (2016). Faecal microbiota transplantation in recurrent Clostridium difficile infection: Recommendations from the French Group of faecal microbiota transplantation. *Digestive and Liver Disease*.

[13] Aas J., Gessert C. E., Bakken J. S. (2003). Recurrent Clostridium difficile colitis: Case series involving 18 patients treated with donor stool administered via a nasogastric tube. *Clinical Infectious Diseases*.

[14] Aratari A., Cammarota G., Papi C. (2015). Fecal microbiota transplantation for recurrent C. difficile infection in a patient with chronic refractory ulcerative colitis. *Journal of Crohn's and Colitis*.

[15] Bilal M., Khehra R., Strahotin C., Mitre R. (2015). Long-term follow-up of fecal microbiota transplantation for treatment of recurrent clostridium difficile infection in a dual solid organ transplant recipient. *Case Reports in Gastroenterology*.

[16] Blackburn L. M., Bales A., Caldwell M., Cordell L., Hamilton S., Kreider H. (2015). Fecal microbiota transplantation in patients with cancer undergoing treatment. *Clinical Journal of Oncology Nursing*.

[17] de Castro C. G., Ganc A. J., Ganc R. L., Petrolli M. S., Hamerschlack N. (2015). Fecal microbiota transplant after hematopoietic SCT: Report of a successful case. *Bone Marrow Transplantation*.

[18] Duplessis C. A., You D., Johnson M., Speziale A. (2012). Efficacious outcome employing fecal bacteriotherapy in severe Crohn's colitis complicated by refractory Clostridium difficile infection. *Infection*.

[19] Ehlermann P., Dösch A. O., Katus H. A. (2014). Donor fecal transfer for recurrent Clostridium difficile-associated diarrhea in heart transplantation. *The Journal of Heart and Lung Transplantation*.

[20] Elopre L., Rodriguez M. (2013). Fecal microbiota therapy for recurrent Clostridium difficile infection in HIV-infected persons. *Annals of Internal Medicine*.

[21] Fischer M., Kao D., Kelly C. (2016). Fecal Microbiota Transplantation is Safe and Efficacious for Recurrent or Refractory Clostridium difficile Infection in Patients with Inflammatory Bowel Disease. *Inflammatory Bowel Diseases*.

[22] Friedman-Moraco R. J., Mehta A. K., Lyon G. M., Kraft C. S. (2014). Fecal microbiota transplantation for refractory Clostridium difficile colitis in solid organ transplant recipients. *American Journal of Transplantation*.

[23] Garborg K., Waagsbø B., Stallemo A., Matre J., Sundøy A. (2010). Results of faecal donor instillation therapy for recurrent Clostridium difficile-associated diarrhoea. *Infectious Diseases*.

[24] Gathe J. C., Diejomaoh E. M., Mayberry C. C., Clemmons J. B. (2016). Fecal Transplantation for Clostridium Difficile - “all Stool May Not Be Created Equal”. *Journal of the International Association of Providers of AIDS Care*.

[25] Gutiérrez-Delgado E. M., Garza-González E., Martínez-Vázquez M. A. (2016). Fecal transplant for Clostridium difficile infection relapses using “pooled” frozen feces from non-related donors. *Acta Gastro-Enterologica Belgica*.

[26] Gweon T.-G., Lee K. J., Kang D. (2015). A case of toxic megacolon caused by *Clostridium difficile* infection and treated with fecal microbiota transplantation. *Gut and Liver*.

[27] Hirsch B. E., Saraiya N., Poeth K., Schwartz R. M., Epstein M. E., Honig G. (2015). Effectiveness of fecal-derived microbiota transfer using orally administered capsules for recurrent Clostridium difficile infection. *BMC Infectious Diseases*.

[28] Hourigan S. K., Chen L. A., Grigoryan Z. (2015). Microbiome changes associated with sustained eradication of *Clostridium difficile* after single faecal microbiota transplantation in children with and without inflammatory bowel disease. *Alimentary Pharmacology & Therapeutics*.

[29] Kelly C. R., Ihunnah C., Fischer M. (2014). Fecal microbiota transplant for treatment of *Clostridium difficile* infection in immunocompromised patients. *American Journal of Gastroenterology*.

[30] Khoruts A., Rank K. M., Newman K. M. (2016). Inflammatory Bowel Disease Affects the Outcome of Fecal Microbiota Transplantation for Recurrent Clostridium difficile Infection. *Clinical Gastroenterology and Hepatology*.

[31] Kronman M. P., Nielson H. J., Adler A. L. (2015). Fecal microbiota transplantation via nasogastric tube for recurrent clostridium difficile infection in pediatric patients. *Journal of Pediatric Gastroenterology and Nutrition*.

[32] Laszlo M., Ciobanu L., Andreica V., Pascu O. (2016). Fecal transplantation indications in ulcerative colitis. preliminary study. *Clujul Medical*.

[33] Lee C. H., Belanger J. E., Kassam Z. (2014). The outcome and long-term follow-up of 94 patients with recurrent and refractory Clostridium difficile infection using single to multiple fecal microbiota transplantation via retention enema. *European Journal of Clinical Microbiology & Infectious Diseases*.

[34] Lee T. J., Jones C. M. (2014). A report of fecal transplantation for refractory clostridium difficile colitis in an orthotopic liver transplant recipient. *Journal of Gastroenterology and Hepatology Research*.

[35] Loke P., Heine R. G., McWilliam V., Cameron D. J. S., Tang M. L. K., Allen K. J. (2016). Fecal microbial transplantation in a pediatric case of recurrent Clostridium difficile infection and specific antibody deficiency. *Pediatric Allergy and Immunology*.

[36] Mandalia A., Ward A., Tauxe W., Kraft C. S., Dhere T. (2016). Fecal transplant is as effective and safe in immunocompromised as non-immunocompromised patients for Clostridium difficile. *International Journal of Colorectal Disease*.

[37] Mattila E., Uusitalo-Seppälä R., Wuorela M. (2012). Fecal transplantation, through colonoscopy, is effective therapy for recurrent *Clostridium difficile* infection. *Gastroenterology*.

[38] Mittal C., Miller N., Meighani A., Hart B. R., John A., Ramesh M. (2015). Fecal microbiota transplant for recurrent Clostridium difficile infection after peripheral autologous stem cell transplant for diffuse large B-cell lymphoma. *Bone Marrow Transplantation*.

[39] Neemann K., Eichele D. D. D., Smith P. P. W., Bociek R., Akhtari M., Freifeld A. (2012). Fecal microbiota transplantation for fulminant *Clostridium difficile* infection in an allogeneic stem cell transplant patient. *Transplant Infectious Disease*.

[40] Ott S. J., Waetzig G. H., Rehman A. (2017). Efficacy of Sterile Fecal Filtrate Transfer for Treating Patients With Clostridium difficile Infection. *Gastroenterology*.

[41] Pathak R., Enuh H. A., Patel A., Wickremesinghe P. (2013). Treatment of relapsing clostridium difficile infection using fecal microbiota transplantation. *Clinical and Experimental Gastroenterology*.

[42] Pierog A., Mencin A., Reilly N. R. (2014). Fecal microbiota transplantation in children with recurrent clostridium difficile infection. *The Pediatric Infectious Disease Journal*.

[43] Porter R. J. (2015). Pulsed faecal microbiota transplantation for recalcitrant recurrent Clostridium difficile infection. *Clinical Microbiology and Infection*.

[44] Quera R., Espinoza R., Estay C., Rivera D. (2014). Bacteremia as an adverse event of fecal microbiota transplantation in a patient with Crohn's disease and recurrent Clostridium difficile infection. *Journal of Crohn's and Colitis*.

[45] Ramay F. H., Amoroso A., Von Rosenvinge E. C., Saharia K. (2016). Fecal microbiota transplantation for treatment of severe, recurrent, and refractory clostridium difficile infection in a severely immunocompromised patient. *Infectious Diseases in Clinical Practice*.

[46] Ray A., Smith R., Breaux J. (2014). Fecal microbiota transplantation for clostridium difficile infection: The ochsner experience. *The Ochsner Journal*.

[47] Rubin T. A., Gessert C. E., Aas J., Bakken J. S. (2013). Fecal microbiome transplantation for recurrent Clostridium difficile infection: Report on a case series. *Anaerobe*.

[48] Russell G. H., Kaplan J. L., Youngster I. (2014). Fecal transplant for recurrent clostridium difficile infection in children with and without inflammatory bowel disease. *Journal of Pediatric Gastroenterology and Nutrition*.

[49] Schünemann M., Oette M. (2014). Fecal microbiota transplantation for Clostridium difficile-associated colitis in a severely immunocompromized critically ill AIDS patient: A case report. *AIDS*.

[50] Silverman M. S., Davis I., Pillai D. R. (2010). Success of Self-Administered Home Fecal Transplantation for Chronic Clostridium difficile Infection. *Clinical Gastroenterology and Hepatology*.

[51] Stripling J., Kumar R., Baddley J. W. (2015). Loss of vancomycin-resistant enterococcus fecal dominance in an organ transplant patient with clostridium difficile colitis after fecal microbiota transplant. *Open Forum Infectious Diseases*.

[52] Trubiano J. A., George A., Barnett J. (2015). A different kind of “allogeneic transplant”: Successful fecal microbiota transplant for recurrent and refractory Clostridium difficile infection in a patient with relapsed aggressive B-cell lymphoma. *Leukemia & Lymphoma*.

[53] Webb B. J., Brunner A., Ford C. D., Gazdik M. A., Petersen F. B., Hoda D. (2016). Fecal microbiota transplantation for recurrent Clostridium difficile infection in hematopoietic stem cell transplant recipients. *Transplant Infectious Disease*.

[54] Weingarden A. R., Hamilton M. J., Sadowsky M. J., Khoruts A. (2013). Resolution of severe clostridium difficile infection following sequential fecal microbiota transplantation. *Journal of Clinical Gastroenterology*.

[55] Yoon S. S., Brandt L. J. (2010). Treatment of refractory/recurrent C. difficile-associated disease by donated stool transplanted via colonoscopy: A case series of 12 patients. *Journal of Clinical Gastroenterology*.

[56] Zainah H., Silverman A. (2012). Fecal bacteriotherapy: A case report in an immunosuppressed patient with ulcerative colitis and recurrent. *Case Reports in Infectious Diseases*.

[57] Study Quality Assessment tools. *Quality Assessment Tool for Case series Studies*.

[58] Ianiro G., Maida M., Burisch J. (2018). Efficiacy of different faecal microbiota transplantation protocols for *Clostridium difficile* infection: A systematic review and meta-analysis. *United European Gastroenterology Journal*.

[59] Brandt L. J., Aroniadis O. C., Mellow M. (2012). Long-term follow-up of colonoscopic fecal microbiota transplant for recurrent *Clostridium difficile* infection. *American Journal of Gastroenterology*.

[60] Bafeta A., Yavchitz A., Riveros C., Batista R., Ravaud P. (2017). Methods and reporting studies assessing fecal microbiota transplantation: A systematic review. *Annals of Internal Medicine*.

[61] Lee C. H., Steiner T., Petrof E. O. (2016). Frozen vs fresh fecal microbiota transplantation and clinical resolution of diarrhea in patients with recurrent clostridium difficile infection a randomized clinical trial. *Journal of the American Medical Association*.

