# Supplementary material for: A Systematic Review of the Efficacy and Safety of Fecal Microbiota Transplant for Clostridium difficile Infection in Immunocompromised Patients
Source: Can J Gastroenterol Hepatol. 2018 Sep 2;2018:1394379. doi: 10.1155/2018/1394379 (PMC6139215; doi:10.1155/2018/1394379)
Supplement: Supplementary 1 — Supplemental File 1: search strategy. [file 1394379.f1.docx]

Supplemental File 1: Search Strategy

| Database | Search String |
| --- | --- |
| Pubmed | (("Clostridium difficile"[Mesh] OR “Clostridium difficile”[tiab] OR “C. diff”[tiab] OR “C. difficile”[tiab] AND ("Fecal Microbiota Transplantation"[Mesh] OR “Fecal Microbiota Transplantation”[tiab] OR “Fecal Microbiota Transplantations”[tiab] OR “Intestinal Microbiota Transfer”[tiab] OR “Intestinal Microbiota Transfers”[tiab] OR “Fecal Transplantation”[tiab] OR “Fecal Transplantations”[tiab] OR “Fecal Transplant”[tiab] OR “Fecal Transplants”[tiab] OR “Donor Feces Infusion”[tiab] OR “Donor Feces Infusions”[tiab] OR “Stool transplant”[tiab] |
| EMBASE | ('Peptoclostridium difficile'/exp OR 'Bacillus difficilis’:ab,ti OR ‘Clostridium difficile':ab,ti OR 'Clostridium difficilis':ab,ti OR ‘C. diff’ OR ‘C difficile’:ab,ti AND ('fecal microbiota transplantation'/exp OR ‘fecal transplant’:ab,ti OR ‘fecal transplantation’:ab,ti OR ‘stool transplant’:ab,ti OR ‘stool transplantation’:ab,ti |
