# Supplementary material for: A Systematic Review of the Efficacy and Safety of Fecal Microbiota Transplant for Clostridium difficile Infection in Immunocompromised Patients
Source: Can J Gastroenterol Hepatol. 2018 Sep 2;2018:1394379. doi: 10.1155/2018/1394379 (PMC6139215; doi:10.1155/2018/1394379)
Supplement: Supplementary 2 — Supplemental File 2: study quality assessment using a modified NIH Quality Assessment Tool. [file 1394379.f2.docx]

Supplemental File 2: Study Quality assessment using a modified NIH Quality Assessment Tool

| Author, Year | Study question/ objective clear | | Case definition | | Cases consecutive | | Subjects comparable | | FMT clearly described | | Outcome measures clearly defined | | Follow-up adequate | | Donor characteristics described | |  |
| --- | --- | --- | --- | --- | --- | --- | --- | --- | --- | --- | --- | --- | --- | --- | --- | --- | --- |
| Aas, 2003 [13] | | Y | | Y | | Y | | N | | Y | | N | | Y | | Y | |
| Garborg, 2010 [23] | | Y | | Y | | Y | | NA | | Y | | Y | | Y | | Y | |
| Hirsch, 2015 [27] | | Y | | Y | | Y | | N | | Y | | Y | | Y | | Y | |
| Hourigan, 2015 [28] | | Y | | Y | | CD | | Y | | Y | | Y | | Y | | Y | |
| Kelly, 2014 [29] | | Y | | Y | | CD | | N | | N | | Y | | Y | | N | |
| Khoruts, 2016 [30] | | Y | | Y | | Y | | Y | | Y | | Y | | Y | | Y | |
| Kronman, 2014 [31] | | Y | | Y | | Y | | N | | Y | | N | | Y | | Y | |
| Laszlo, 2016 [32] | | Y | | Y | | N | | Y | | Y | | Y | | Y | | Y | |
| Lee CH, 2014 [34] | | Y | | Y | | Y | | N | | Y | | Y | | Y | | Y | |
| Mandalia, 2016 [36] | | Y | | Y | | CD | | N | | Y | | Y | | Y | | Y | |
| Mittal, 2015 [38] | | Y | | Y | | CD | | N | | Y | | Y | | Y | | Y | |
| Ott, 2017 [40] | | Y | | N | | CD | | N | | Y | | N | | Y | | Y | |
| Pathak, 2014 [41] | | Y | | Y | | Y | | N | | Y | | Y | | Y | | Y | |
| Pierog, 2014 [42] | | Y | | Y | | CD | | N | | Y | | Y | | CD | | Y | |
| Ray, 2014 [46] | | Y | | Y | | CD | | N | | Y | | N | | Y | | Y | |
| Rubin, 2012 [47] | | Y | | Y | | Y | | N | | Y | | Y | | Y | | Y | |
| Russell, 2014 [48] | | Y | | Y | | CD | | N | | Y | | N | | Y | | Y | |
| Silverman, 2010 [50] | | Y | | N | | CD | | N | | Y | | N | | Y | | Y | |
| Webb, 2016 [53] | | Y | | Y | | Y | | Y | | Y | | N | | Y | | Y | |
| Yoon, 2010 [55] | | Y | | Y | | Y | | N | | Y | | Y | | CD | | Y | |

Y=yes; N=no; CD=cannot determine; NA=not applicable

NIH Quality Assessment Tool for Case Series Studies*

1. Was the study question or objective clearly stated?

2. Was the study population clearly and fully described, including a case definition?

3. Were the cases consecutive?

4. Were the subjects comparable?

5. Was the intervention (FMT) clearly described?

6. Were the outcome measures clearly defined?

7. Was the length of follow up adequate?

8. Donor characteristics described

*This is a modified version with Question 8 (Donor characteristic added in and questions 8 and 9 of original version removed)
